# Role of Macrophage lncRNAs in Mediating Inflammatory Processes in Atherosclerosis and Sepsis

**DOI:** 10.3390/biomedicines11071905

**Published:** 2023-07-05

**Authors:** Hyeung-Seob Shin, Jae-Joon Shin, Jeongkwang Park, Imene Arab, Kyoungho Suk, Won-Ha Lee

**Affiliations:** 1 BK21 Plus KNU Creative BioResearch Group, School of Life Sciences, Kyungpook National University, Daegu 41566, Republic of Korea; gudals3963@naver.com (H.-S.S.); wowns1124@knu.ac.kr (J.-J.S.); wjdrhkd28787@gmail.com (J.P.); arabimene07@gmail.com (I.A.); 2 Department of Pharmacology, Brain Science & Engineering Institute, BK21 Plus KNU Biomedical Convergence Program, School of Medicine, Kyungpook National University, Daegu 41944, Republic of Korea; ksuk@knu.ac.kr

**Keywords:** atherosclerosis, inflammation, lncRNA, macrophage, sepsis, therapeutic targets

## Abstract

Long noncoding RNAs (lncRNAs) are molecules >200 bases in length without protein-coding functions implicated in signal transduction and gene expression regulation via interaction with proteins or RNAs, exhibiting various functions. The expression of lncRNAs has been detected in many cell types, including macrophages, a type of immune cell involved in acute and chronic inflammation, removal of dead or damaged cells, and tissue repair. Increasing evidence indicates that lncRNAs play essential roles in macrophage functions and disease development. Additionally, many animal studies have reported that blockage or modulation of lncRNA functions alleviates disease severity or morbidity rate. The present review summarizes the current knowledge regarding lncRNAs expressed in macrophages, focusing on their molecular targets and the biological processes regulated by them during the development of inflammatory diseases such as atherosclerosis and sepsis. Possible application of this information to lncRNA-targeting therapy is also discussed. The studies regarding macrophage lncRNAs described in this review can help provide valuable information for developing treatments for various pathological conditions involving macrophages.

## 1. Introduction

Recent advances in molecular biology have revealed that although noncoding RNAs (ncRNAs) are not translated into proteins, they play various roles in cellular processes and disease pathogeneses. Long ncRNAs (lncRNAs) are >200 nucleotides in length and have been extensively researched in various fields of biology [1]. Nuclear lncRNAs have been implicated in regulating chromatin organization, gene transcription, RNA splicing, and epigenetic modifications [2,3,4,5]. Certain lncRNAs that exhibit structural features similar to those of mRNAs can be transported to the cytoplasm to modulate signaling pathways and post-transcriptional gene expression regulation by affecting mRNA stability and translation or sponging microRNAs (miRNAs) to block their function [6,7,8,9,10].

Macrophages exhibit immunoregulatory functions during acute and chronic inflammation, pathogenesis of various diseases, and cancer development. They are categorized into M1 and M2 functional groups. M1 macrophages (also known as “killer” or classically activated macrophages) phagocytose pathogens and foreign substances and promote inflammation, whereas M2 macrophages (also known as “repair” or alternatively activated macrophages) mediate tissue repair and inflammation resolution [11,12]. M1 macrophages metabolize arginine to nitric oxide and synthesize ATP via glycolysis. Furthermore, the mitochondrial citric acid cycle is shut down in these cells. Conversely, M2 macrophages metabolize arginine into ornithine or proline and mostly synthesize ATPs via the citric acid cycle [13]. The bacterial endotoxin lipopolysaccharide (LPS) and interferon (IFN)-γ induce the differentiation of undifferentiated (M0) macrophages into M1 macrophages, which produce a range of proinflammatory cytokines, including tumor necrosis factor (TNF)-α, interleukin (IL)-1, and IL-12. These cytokines and M1 macrophages have been associated with acute inflammation and tissue damage. Conversely, M2 differentiation, induced by IL-4 and IL-13, is characterized by the production of anti-inflammatory cytokines, such as IL-10 and transforming growth factor (TGF)-β. These cytokines and M2 macrophages help regulate immune responses and promote tissue repair [13,14]. Given that the activation and differentiation pathways and immunological roles of M1 and M2 macrophages differ, a detailed understanding of the factors that maintain or regulate the balance between them is important to effectively treat various diseases, such as autoimmune diseases, inflammatory bowel diseases, diabetes, obesity, rheumatoid arthritis (RA), and systemic sclerosis [13,15].

With the identification of an increasing number of lncRNAs involved in regulating macrophage activities and maintaining M1 and M2 polarization balance, lncRNAs are expected to become effective therapeutic targets for various diseases. Previous studies have extensively summarized the implications of lncRNAs regulating inflammatory diseases and macrophage M1 and M2 polarization [16,17]. Herein, we investigated the roles of recently identified and/or salient macrophage lncRNAs in the pathogenesis of atherosclerosis and sepsis, two diseases in which macrophage-mediated inflammation plays an important role.

## 2. Atherosclerosis

Atherosclerosis is a chronic inflammatory disease characterized by the narrowing and hardening of arteries due to the accumulation of lipid-laden plaques on their inner walls. Macrophages engulf modified low-density lipoprotein (LDL) particles, such as oxidized LDL (oxLDL), and differentiate into foam cells, thereby aggravating chronic inflammatory conditions, stimulating plaque growth, and destabilizing plaques [18,19]. Atherosclerosis has been associated with various health problems, including coronary artery diseases (CADs) and stroke. M1 and M2 macrophages have been implicated in atherogenesis. M1 macrophages promote inflammation and plaque rupture by producing proinflammatory cytokines, chemokines, reactive oxygen species (ROS), and extracellular matrix-degrading enzymes. Conversely, M2 macrophages resolve inflammation by releasing anti-inflammatory cytokines, such as IL-10 [13,20,21]. Most studies on the role of macrophage lncRNAs in atherosclerosis have focused on the regulation of proinflammatory activities, oxLDL-mediated lipid accumulation, cholesterol efflux, and foam cell formation, as described below.

**Figure 1:**
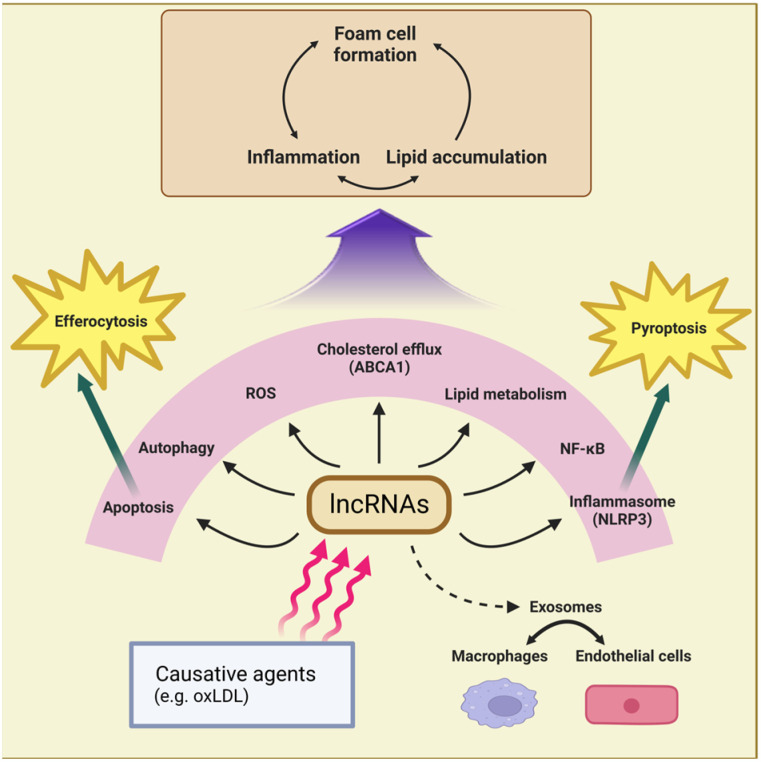
Action mechanisms of macrophage lncRNAs involved in atherogenesis. These lncRNAs affect various cellular processes that ultimately regulate inflammation, lipid accumulation, and foam cell formation.

**Figure 2:**
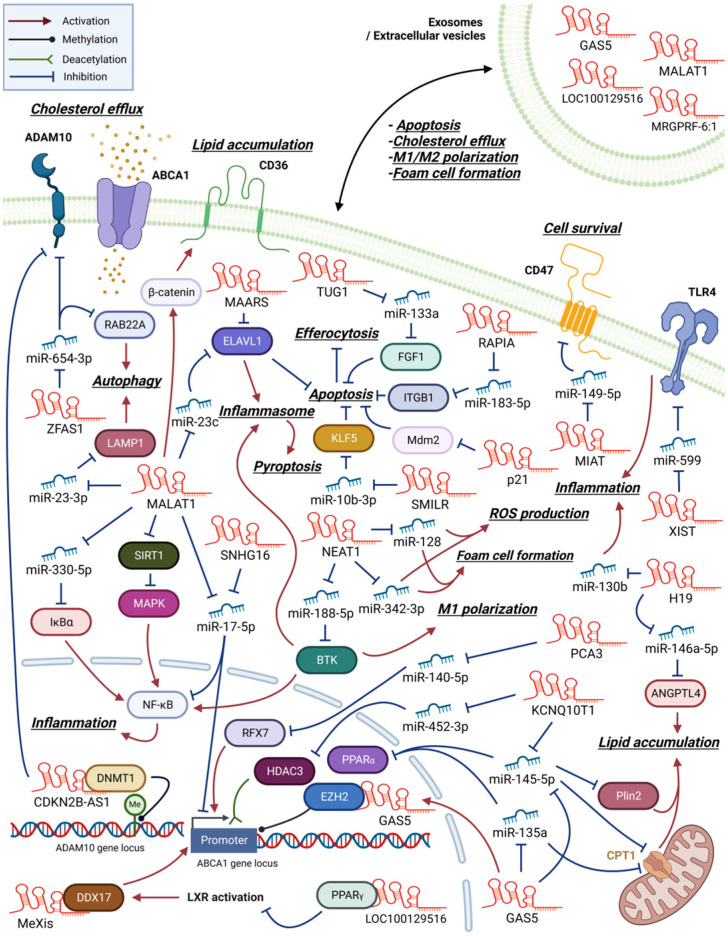
An overview of macrophage lncRNAs involved in atherosclerosis development. LncRNAs exert their functions through both direct and indirect mechanisms. Certain lncRNAs act as miRNA sponges, indirectly regulating protein expression by inhibiting miRNA function. In contrast, other lncRNAs directly interact with proteins to modulate their activity. Additionally, some lncRNAs can regulate gene expression through epigenetic modifications. Within the figures, arrows are employed to indicate the molecule responsible for activating or inhibiting its specific target.

Numerous lncRNAs have been linked to atherogenesis, and these lncRNAs are categorized and representative ones are described in the text. Table 1 lists the macrophage lncRNAs that regulate atherogenesis, their targets, and the cellular functions affected by them. Figure 1 summarizes the functions performed by these lncRNAs, and Figure 2 illustrates the action mechanisms of representative lncRNAs.

### 2.1. LncRNAs That Promote Inflammation and Foam Cell Formation

Many macrophage lncRNAs involved in atherosclerosis development are induced by oxLDL and promote foam cell formation through enhancing inflammatory changes (Figure 2). Previous studies found that lncRNA H19 expression was upregulated after oxLDL treatment in peripheral blood mononuclear cells of patients with CAD, plaque macrophages of an atherosclerotic mouse model, and RAW264.7 murine macrophage-like cells [22,23]. Transfecting H19-specific short hairpin RNA (shRNA) into RAW264.7 cells decreased oxLDL-induced lipid accumulation and proinflammatory mediator expression by regulating miR-130b activity [24]. Additionally, H19 facilitated lipid accumulation in macrophages via sponging miR-146a-5p and, consequently, protecting angiopoietin-like 4 (ANGPTL4) (miR-146a-5p/ANGPTL4 axis) [23]. ANGPTL4 is a released glycoprotein that regulates lipid metabolism and insulin sensitivity; thus, alterations in ANGPTL4 expression affect the risk of developing atherosclerosis and type 2 diabetes mellitus [25]. 

NEAT1 (LINC00084, shortened from either nuclear paraspeckle assembly transcript 1 or nuclear enriched abundant transcript 1) is a nucleus-restricted lncRNA involved in the formation of paraspeckles, which are subnuclear structures implicated in antiviral responses [26]. NEAT1 expression is upregulated in oxLDL-treated THP-1 cells, a human monocytic leukemia cell line with macrophage-like properties. Furthermore, NEAT1 participates in the formation of paraspeckles and the development of subsequent proinflammatory responses by regulating p65 phosphorylation. Additionally, NEAT1 modulates lipid uptake by regulating the expression of a scavenger receptor, CD36 [27]. The treatment of RAW264.7 cells with oxLDL increases NEAT1 expression, which in turn stimulates proinflammatory cytokine and ROS production, subsequently promoting foam cell formation by sponging miR-128 [28]. Similar results were observed in oxLDL-treated THP-1 cells; however, the target of NEAT1 action was miR-342-3p [29]. Treating BMDMs with titanium particles induces the expression of NEAT1, which then sponges miR-188-5p to stabilize Bruton’s tyrosine kinase (BTK) mRNA. This NEAT1/miR-188-5p/BTK axis eventually promotes the activation of NF-κB, formation of the NLR family pyrin domain containing 3 (NLRP3) inflammasome, and M1 polarization [30]. Thus, NEAT1 expression in activated macrophages enhances proinflammatory changes. However, one contradicting study reported decreased NEAT1 levels in peripheral blood mononuclear cells (PBMCs) of post-myocardial infarction (MI) patients and demonstrated enhanced inflammatory activation of macrophages in *NEAT1*-knockout mice [31]. 

In THP-1 cells, oxLDL treatment upregulated lncRNA urothelial cancer-associated 1 (UCA1) expression levels, which further exacerbated atherosclerotic events, such as CD36 expression, foam cell formation, and ROS generation via sponging miR-206 [32]. In atherosclerotic animal models, lncRNA dynamin 3 opposite strand (Dnm3os) expression was increased in atherosclerotic plaques. Dnm3os regulated macrophage proinflammatory activities via the miR-27b-3p/signaling lymphocytic activation molecule 7 (SLAMF7) axis [33]. SLAMF7 is a membrane protein whose expression is upregulated in macrophages during phagocytosis and macrophage differentiation in atherosclerotic plaques [34].

### 2.2. LncRNAs That Regulate Cholesterol Efflux and Foam Cell Formation

Numerous clinical and animal studies have demonstrated that defects in reverse cholesterol transport and cholesterol efflux are associated with an increased risk of cardiovascular diseases and atherosclerosis [18,19]. ATP-binding cassette subfamily A member 1 (ABCA1)-mediated cholesterol efflux reduces the formation of lipid-laden foam cells in atherosclerotic plaques. Because regulation of cholesterol efflux is crucial for the prevention of atherosclerosis, ABCA1 has been one of the primary targets in lncRNA research. Interestingly, most of these lncRNAs are involved in regulating chromatin activity and the transcription of the *ABCA1* gene, as described below.

The lncRNA macrophage-expressed liver X receptor (LXR)-induced sequence (MeXis) is reportedly involved in LXR-dependent transcriptional activation of *Abca1* by guiding the promoter binding of the transcription coactivator DEAD-box helicase 17 (DDX17). Furthermore, bone marrow cells from *MeXis*-deficient mice exhibited altered chromosome architecture at the *Abca1* locus, impaired cholesterol efflux, and accelerated atherosclerosis development. Notably, the genes encoding *ABCA1* and *MeXis* are located near one another to ensure tissue-selective activation of this regulatory circuit [35]. Prostate cancer antigen 3 (PCA3), another lncRNA, also promoted ABCA1 expression by activating the transcription activator RFX7 through sponging miR-140-5p [36]. In the case of HAND2 antisense RNA 1 (HAND2-AS1), ABCA1 expression was promoted by sponging miR-128 and subsequently activating the class II histone deacetylase, sirtuin 1 (SIRT1) [37].

In contrast, the lncRNA growth arrest-specific 5 (GAS5) exerts inhibitory effects on ABCA1 function through its interaction with and stabilization of the enhancer of zeste homolog 2 (EZH2), a chromatin-repressive complex known to promote trimethylation of lysine 27 (H3K27) at the *Abca1* promoter [38]. Notably, a significant elevation in GAS5 levels was detected in the serum of patients with coronary heart disease, exhibiting a correlation with heightened proinflammatory markers [39]. Similarly, the lncRNA Kcnq1 overlapping transcript 1 (KCNQ1OT1) impedes ABCA1-mediated cholesterol efflux by suppressing chromatin activity at the *Abca1* locus via the miR-452-3p/histone deacetylase 3 (HDAC3) axis [40]. Intriguingly, lncRNA AI662270 directly binds to ABCA1, exerting a limiting effect on its function and thereby promoting lipid accumulation and foam cell formation [41]. Consistent with expectations, animal models have demonstrated that lncRNAs promoting ABCA1 expression alleviate atherosclerosis, whereas those suppressing ABCA1 expression exacerbate its progression [36,38,40].

### 2.3. LncRNAs That Regulate Macrophage Apoptosis, Pyroptosis, or Autophagy in Atherosclerosis

Cellular processes such as apoptosis, pyroptosis, and autophagy need to be balanced with macrophage proliferation. Disruption of the balance may destabilize atherosclerotic plaques. Apoptotic cell death, especially in overstimulated or exhausted foam cells, can enhance inflammation and trigger blood clot formation, which may lead to heart attack or stroke [18,19]. 

**Table 1:** Macrophage lncRNAs involved in atherosclerosis development. The grouping is arbitrary and lncRNA in one group may have roles that are relevant to other categories.

LncRNA	Cell Type	Target	Affected Function	Ref.
Group 1. LncRNAs that regulate inflammation and foam cell formation				
H19	-	miR-130b	Modulates cell survival, lipid accumulation, and inflammatory response	[24]
H19	Foam cell	miR-146a-5p/ANGPTL4	ox-LDL-induced, regulates lipid accumulation, accelerates foam cell formation	[23]
Dnm3os	-	miR-27b-3p/SLAMF7	Streptosotocin-induced, intermedin-repressed, participates in ox-LDL absorption	[33]
KCNQ10T1	-	miR-145-5p/PPARα	ox-LDL-induced, participates in lipid metabolism and inflammation	[42]
MALAT1	Foam cell	miR-330-5p/IκBα	Activates NF-κB pathway, enhances lipid accumulation and inflammation	[43]
MALAT1	-	-	*MALAT1*-deficient *apoE*^−/−^ mice exhibit enhanced inflammation and atherosclerosis	[44]
MALAT1	Foam cell	β-catenin/CD36	ox-LDL-induced, induces lipid uptake	[45]
MALAT1	Foam cell	SIRT1/MAPK/NF-κB	ox-LDL-induced, inhibits activation of NF-κB pathway	[46]
NEAT1	-	NONO and p65	ox-LDL-induced, mediates paraspeckle formation, regulates lipid uptake	[27]
NEAT1	M1	miR-188-5p or KLF4/BTK	Activates NLRP3 inflammasome and NF-κB pathway, induces M1 polarization	[30]
NEAT1	Foam cell	miR-128	Facilitates inflammation, oxidative stress responses and foam cell formation	[28]
NEAT1	-	miR-342-3p	ox-LDL-induced, regulates inflammation and lipid uptake	[29]
NEXN-AS1	-	BAZ1A/NEXN	Attenuates adhesion activity of macrophage, suppresses inflammatory gene expression	[47]
SNHG16	Foam cell	miR-17-5p/NF-κB	ox-LDL-induced, enhances cell proliferation and inflammatory responses	[48]
UCA1	Foam cell	miR-206	ox-LDL-induced, facilitates foam cell formation, regulates ROS levels	[32]
Group 2. LncRNAs that regulate cholesterol efflux and foam cell formation				
AI662270	Foam cell	ABCA1	ox-LDL-induced, suppresses cholesterol efflux, enhances lipid accumulation	[41]
CDKN2B-AS1	Foam cell	DNMT1/ADAM10	Promotes cholesterol efflux, reduces inflammatory responses	[49]
GAS5	Foam cell	EZH2/ABCA1	Inhibits cholesterol efflux, enhances lipid accumulation and atherogenesis	[38]
HAND2-AS1	Foam cell	miR-1208/SIRT1/ABCA1	Inhibits foam cell formation, accelerates cholesterol efflux, attenuates atherosclerosis	[37]
KCNQ1OT1	-	miR-452-3p/HDAC3/ABCA1	ox-LDL-induced, inhibits cholesterol efflux, facilitates foam cell formation	[40]
MALAT1	Foam cell	miR-17-5p/ABCA1	Decreases in ox-LDL induced THP-1 derived macrophage, inhibits cholesterol efflux	[50]
MeXis	Foam cell	DDX17/ABCA1	LXR-induced, remodels chromatin at the target locus, induces cholesterol efflux	[35]
PCA3	Foam cell	miR-140-5p/RFX7ABCA1	promotes cholesterol efflux, inhibits atherosclerosis progression	[36]
TUG1	Foam cell	miR-92a/FXR1	Regulates apolipoprotein M, downregulates cholesterol efflux, aggravates atherosclerosis	[51]
ZFAS1	Foam cell	miR-654-3p/ADAM10 miR-654-3p/RAB22A	ox-LDL-induced, activates inflammation, inhibits cholesterol efflux	[52]
Group 3. LncRNAs that regulate apoptosis, pyroptosis, and autophagy				
ANRIL	-	Alu repeats etc.	Increases proliferation, enhances metabolic activity, decreases apoptosis	[53]
GAS5	Foam cell	miR-145-5p/Plin2	Facilitates oxLDL uptake and autophagy, accelerates foam cell formation	[54]
MIAT	-	miR-149-5p/CD47	ox-LDL-induced, inhibits efferocytosis of macrophage, accelerates atherogenic process	[55]
MALAT1	-	miR-23c/ELAVL1	ox-LDL-induced, activates NLRP3 inflammasome mediate pyroptosis	[56]
MAARS	-	ELAVL1(HuR)	Activates HuR target gene expression, promotes apoptosis, decreases efferocytosis	[57]
MALAT1	-	miR-23-3p/LAMP1	Rapa induced, facilitates autophagy activity	[58]
p21	-	Mdm2	Inhibits cell proliferation, induces apoptosis, regulates p300/p53 interaction	[59]
TUG1	-	miR-133a/FGF1	Facilitates cell proliferation, activates inflammation, inhibits apoptosis	[60]
XIST	-	miR-599/TLR4	ox-LDL-induced, inhibits apoptosis, aggravates atherosclerosis progression	[61]
RAPIA	-	miR-183-5p/ITGB1	Induced by the action of FoxO1, activates cell proliferation, inhibits apoptosis	[62]
SMILR	-	miR-10b-3p/KLF5	ox-LDL-induced, activates cell proliferation, inhibits apoptosis	[63]
Group 4. LncRNAs that acts through exosomes				
GAS5	-	p53	Produced by macrophages, induces apoptosis in macrophages and vascular endothelial cells	[64]
MALAT1	M2	-	Exported via ox-LDL-induced HUVEC-derived exosome, induces M2 polarization	[65]
MALAT1	M1	miR-25-3p/CDC42	Affects endothelial cells. Inhibits angiogenesis and myocardial regeneration, aggravates MI	[66]
MRGPRF-6:1	M1	TLR4/MyD88/MAPK	Enhances foam cell formation and M1 polarization. Detected in the plasma exosomes	[67]
LOC100129516	Foam cell	PPARγ/LXRα/ABCA1	MSC-derived exosomes, inhibits foam cell cholesterol efflux, aggravates atherosclerosis	[68]

LncRNAs that inhibit apoptosis tend to aggravate atherogenesis [53,60,61,62,63]. By suppressing apoptosis, macrophages are allowed to proliferate and promote plaque formation. In addition to inhibiting apoptosis, lncRNAs such as taurine-upregulated gene 1 (TUG1) and X-inactive specific transcript (XIST) enhance inflammation via fibroblast growth factor 1 and Toll-like receptor 4 (TLR4), respectively [60,61]. LncRNAs associated with the progression and intervention of atherosclerosis (RAPIA) and smooth-muscle-induced lncRNA (SMILR) also inhibit apoptosis and enhance atherosclerosis by regulating cellular receptor integrin beta 1 (ITGB1) and a transcription factor, Krueppel-like factor 5 (KLF5), respectively [62,63]. In contrast, apoptosis-inducing lncRNAs such as lncRNA-p21 have an atheroprotective role by reducing macrophage proliferation and consequent inflammation [59]. These effects of apoptosis-regulating lncRNAs on atherogenesis have been confirmed in animal models in the cases of p21 and TUG1 [59,60].

The progression of atherosclerosis may also be aggravated by defects in a process known as efferocytosis, the clearance of apoptotic cells by macrophages. Simion et al., detected high-level expression of a macrophage-associated atherosclerotic lncRNA sequence (MAARS) in the aortic intima of atherogenic animal models, and it decreased with the regression of atherosclerosis. Knockdown experiments indicated that MAARS promotes macrophage apoptosis, thereby inhibiting efferocytosis, through its interaction with HuR (ELAVL1), an RNA-binding protein with an apoptosis regulator function [57]. Another lncRNA, MI-associated transcript (MIAT), directly affects efferocytosis [55]. Its expression has been detected in the serum of patients with advanced atherosclerosis and necrotic core macrophages. In RAW264.7 cells, treatment with oxLDL increases MIAT levels. MIAT prevents efferocytosis by sponging miR-149-5p and, consequently, increasing the expression of the antiphagocytic molecule CD47. Suppressing MIAT expression in experimental animal models attenuated atherosclerosis progression by reducing the necrotic core size and increasing plaque stability.

Autophagy and inflammasome-mediated pyroptotic cell death in macrophages may also affect plaque stability, and GAS5 and MALAT1 have been reported to be involved in these processes (Table 1).

### 2.4. LncRNAs Functioning via Exosomes in Atherogenesis

Many studies have indicated that exosomes can be used as carriers of lncRNA to regulate cellular activities of neighboring cells. The upregulation of lnc-MRGPRF-6:1 expression and as its correlation with levels of proinflammatory mediators have been detected in the plasma exosomes of patients with CAD [67]. The expression level of lnc-MRGPRF-6:1 following M1 induction was higher than that following M2 induction in THP-1 cells. The knockout of *lnc-MRGPRF-6:1* reduced ROS generation, lipid accumulation, and subsequent foam cell formation. Furthermore, *lnc-MRGPRF-6:1* knockout in human monocyte-derived macrophages suppressed M1 marker and inflammatory cytokine expression and enhanced M2 marker expression by modulating the TLR4/myeloid differentiation primary response 88 (MyD88)/mitogen-activated protein kinase (MAPK) signaling pathway [67]. 

Notably, exosomes derived from mesenchymal stem cells (MSCs) have been developed as a method for drug delivery for therapeutic purposes [69]. Delivery of LOC100129516-specific siRNA using MSC-derived exosomes blocked cholesterol efflux in foam cells by suppressing the LOC100129516/PPARγ/LXRα/ABCA1 axis [68]. The overexpression of *GAS5* in THP-1 cells enhanced oxLDL-induced apoptosis. The resulting apoptotic cells further secreted GAS5-containing exosomes, thereby enhancing apoptosis in vascular endothelial cells [64]. 

### 2.5. Multiple Function of MALAT1 in Atherogenesis

The lncRNA metastasis-associated lung adenocarcinoma transcript 1 (MALAT1) affects multiple atherosclerotic processes, such as foam cell formation and macrophage apoptosis, autophagy, and pyroptosis (Figure 2). Treating THP-1 cells with oxLDL upregulated MALAT1 expression in a nuclear factor kappa-light-chain-enhancer of activated B cell (NF-κB)-dependent manner [43,45]. MALAT1 enhanced lipid uptake by inducing CD36 expression by recruiting β-catenin to its binding sites on the CD36 promoter [45]. MALAT1 also enhanced NF-κB activation and, subsequently, foam cell formation, apoptosis, and inflammation via sponging miR-330-5p [43]. Additionally, MALAT1 promoted autophagy in RAW264.7 cells by modulating the miR-23-3p/lysosomal-associated membrane protein 1 (LAMP1) axis [58]. LAMP1 plays a key role in the fusion of autophagosomes with lysosomes. In THP-1 cells, MALAT1 mediated oxLDL-induced autophagy by inhibiting sirtuin 1, a deacetylase enzyme regulating various transcription factors and enzymes, and activating the MAPK/NF-κB pathway [46]. These effects of MALAT1 have been confirmed in an atherosclerotic mouse model, wherein *MALAT1* overexpression enhanced disease severity [43]. Treating diabetic atherosclerosis rat models with sinapic acid, a derivative of cinnamic acid, improved rat body weight and reduced their blood glucose levels. Bone-marrow-derived macrophages (BMDMs) isolated from disease animal models treated with low doses of sinapic acid exhibited less inflammasome activation and pyroptotic cell death [56]. In vitro analyses revealed that these beneficial effects of sinapic acid originate from its ability to suppress the expression of MALAT1, which promotes pyroptotic cell death in macrophages and, consequently, the progression of atherosclerosis. Pyroptosis is an inflammasome-mediated programmed cell death that occurs as a defense mechanism against intracellular pathogens. MALAT1 was also found in extracellular vesicles (EVs) derived from M1 BMDMs. These EVs affect myocyte proliferation and angiogenesis in MI animal models [66]. Thus, the findings of these studies indicate that MALAT1 expression is upregulated in macrophages by stimulating agents, such as oxLDL, and MALAT1 enhances atherosclerosis by inducting lipid uptake, foam cell formation, and cell death in macrophages.

However, there are reports of an opposite role played by MALAT1 in atherosclerosis. For instance, in an apolipoprotein E (*apoE*)-knockout mouse model, *MALAT1* deficiency accelerated inflammation and atherosclerosis. Treating *MALAT1*-deficient BMDMs with LPS enhanced TNF-α and inducible nitric oxide synthase expression, suppressed matrix metalloproteinase-9 expression, and impaired phagocytic activity [44]. Furthermore, decreased MALAT1 levels have been detected in the serum of patients with atherosclerosis and in oxLDL-treated THP-1 cells. MALAT1 knockdown in THP-1 cells increased oxLDL uptake, lipid accumulation, and total cholesterol levels by regulating the miR-17-5p/ABCA1 axis [50]. Notably, treating THP-1 cells with exosomal MALAT1 derived from oxLDL-treated human umbilical vein endothelial cells (HUVECs) suppressed M1 marker expression and enhanced M2 marker expression [65]. These results suggest that suppressing or downregulating MALAT1 expression accelerates atherosclerosis progression. The reason for the discrepancy in the role of MALAT1 in atherosclerosis remains unknown; thus, additional analyses are warranted.

## 3. Sepsis

**Figure 3:**
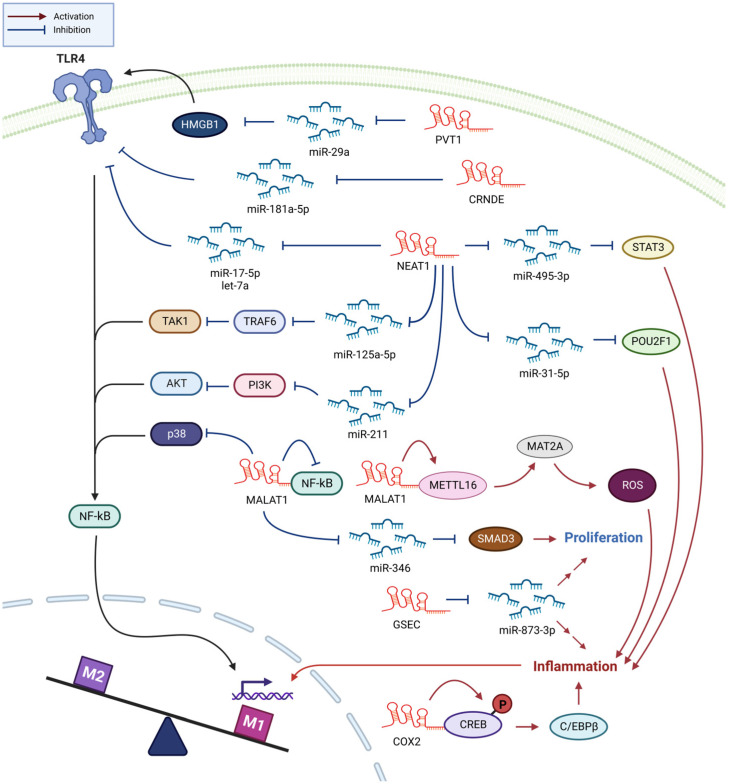
Action mechanisms of macrophage lncRNAs involved in sepsis development. Most of these lncRNAs are involved in LPS-induced inflammatory activation and subsequent M1 polarization and proliferation of macrophages.

Blood monocytes/macrophages and endothelial cells lining the blood vessels respond to gram-negative bacteria infiltration by releasing a flood of chemicals, including cytokines, into circulation to fight the infection. Macrophages can remove pathogens by phagocytosis and regulate the extent of sepsis by producing anti-inflammatory cytokines. However, the production of excess inflammatory cytokines, such as IL-6, IL-1β, and especially TNF-α, may damage the surrounding normal tissues and organs, which can be life-threatening [70,71]. As observed in other diseases, the proinflammatory activity of M1 macrophages aggravates sepsis, whereas the anti-inflammatory activity of M2 macrophages mitigates it [72]. Most studies regarding the role of macrophage lncRNAs in sepsis have focused on the proinflammatory effects and M1/M2 polarization of macrophages, as described below and summarized in Figure 3.

### 3.1. NEAT1 Enhances Sepsis Progression through Promoting Inflammation

Previous studies have found a considerable increase in NEAT1 levels in serum of patients with sepsis and septic mouse models [73,74,75,76]. These studies agree that NEAT1 is involved in the inflammatory activation of macrophages; however, the targets of its action differ. In THP-1 cells, LPS-induced NEAT1 expression enhances inflammatory responses by modulating the miR-17-5p/TLR4 axis [76]. LPS-stimulated Kupffer or RAW264.7 cells exhibit the expression of NEAT1, which exerts its proinflammatory activities through the Let-7q/TLR4 axis [73]. Other studies have reported that NEAT1 promotes inflammation in LPS-treated RAW264.7 cells by modulating the miR-495-3p/signal transducer and activator of transcription 3 (STAT3), miR-211/phosphoinositide 3-kinase (PI3K)/protein kinase B (AKT), miR-370-3p/thrombospondin-1, or miR-31-5p/POU domain, class 2, transcription factor 1 (POU2F1) axes [74,75,77]. Wang et al., reported that this effect of NEAT1 was mediated through the miR-125a-5p/TNF receptor-associated factor 6 (TRAF6)/TGF-β-activated kinase 1 (TAK1) axis and that the downregulation of NEAT1 expression promoted M2 polarization [78]. Thus, NEAT1 expressed in activated macrophages enhances their proinflammatory activity. As most previous experiments have been performed using cell lines, additional studies involving animal models or primary macrophages isolated from patients with sepsis are warranted to confirm the role of NEAT1 in sepsis progression.

### 3.2. MALAT1 Promotes M1 Polarization and Inflammation in Sepsis

An increase in lncRNA MALAT1 levels was detected in the serum of late-onset sepsis patients and in activated primary macrophages and macrophage cell lines [79]. *MALAT1*-knockout mice exhibited reduced inflammation and death upon sepsis induction. Particularly, suppressing MALAT1 expression increased the antioxidant capacity of macrophages through the methyltransferase 16 (METTL16)/methionine adenosyltransferase 2 A (MAT2A) axis, wherein MALAT1 binds to METTL16, thereby stabilizing the METTL16 N6-methyladenosine (m6A) modification activity [79]. MAT2A regulates cellular metabolism and catalyzes S-adenosylmethionine production [80]. Intraperitoneal LPS injection in mice induces septic lung injury, substantially increasing MALAT1 expression in lung tissues. Additional intravenous MALAT1-specific small interfering RNA (siRNA) injection reduces the number of inflammatory cells and cytokine levels in the bronchoalveolar lavage fluid (BALF) of these animal models by inhibiting the p38 MAPK/p65 NF-κB signaling pathway [81]. Cui et al., reported that LPS and IL-4 treatments increased and decreased MALAT1 expression levels, respectively, in human and mouse macrophage cell lines and primary macrophages. Attenuated MALAT1 expression suppresses M1 and enhances M2 polarization in primary macrophages. Although *MALAT1*-knockout mice exhibit attenuated symptoms of LPS-induced acute lung injury, profibrotic macrophage differentiation and pulmonary fibrosis are promoted in these mice [82]. Thus, macrophage MALAT1 levels increase during LPS-induced macrophage activation and sepsis development. MALAT1 also mediates proinflammatory changes, further implicating MALAT1 as an attractive therapeutic target of sepsis.

However, a few studies have reported different observations regarding the role of MALAT1. For instance, Yang et al., reported a significant decrease in MALAT1 serum levels and an increase in hsa-miR-346 levels in patients with sepsis. Activated RAW264.7 cells also exhibit reduced expression of MALAT1. Additional experiments demonstrated that MALAT1 regulates macrophage proliferation through the hsa-miR-346/small mothers against decapentaplegic homolog 3 (SMAD3) axis [83]. SMAD3 is a receptor-regulated signaling adaptor activated by serine kinases. Zhao et al., demonstrated that LPS treatment upregulated MALAT1 expression in human and mouse macrophage-like cell lines and primary macrophages. This increase in MALAT1 expression levels is NF-κB dependent, and MALAT1 suppresses the expression of proinflammatory cytokines, such as TNF-α and IL-6, by interacting with NF-κB to block its DNA-binding activity [84]. The cause of this discrepancy in the role of macrophage MALAT1 in sepsis development is currently unknown, and more research on this subject will aid in reaching a definitive conclusion.

### 3.3. Other lncRNAs Involved in Sepsis Development

LPS-induced NF-κB activation in THP-1 cells and the subsequent release of proinflammatory cytokines were shown to be regulated by lncRNA colorectal neoplasia differentially expressed (CRNDE) via the miR-181-5p/TLR4 axis. A considerable increase in CRNDE expression levels and decrease in miR-181-5p expression levels have been detected in the peripheral blood of patients with sepsis. Furthermore, the extent of these changes correlates with the survival rate of the patients [85]. In sepsis patients, the serum levels of G-quadruplex-forming sequence-containing lncRNA (GSEC) were observed to be elevated, and GSEC was identified as a regulator of inflammation and proliferation in RAW264.7 cells through its interaction with miR-873-3p [86]. Conversely, sepsis patients exhibited a significant decrease in serum levels of lncRNA MEG3. In vitro analysis demonstrated that *MEG3* overexpression suppressed macrophage apoptosis and NF-κB-mediated inflammation. Notably, in a septic mouse model, silencing of lncRNA Cox2 resulted in the amelioration of sepsis symptoms by suppressing M1 and enhancing M2 macrophage activities [87]. Moreover, elevated expression of lncRNA PVT1 was detected in heart-infiltrating macrophages of septic mice, and PVT1 was found to enhance M1 polarization through the miR-29a/HMGB1 axis [88].

## 4. Discussion and Conclusions 

Macrophage lncRNAs are induced by causative agents, such as oxLDL, and affect atherogenesis by regulating a wide range of processes including NF-κB activation, ROS generation, cholesterol efflux, apoptosis, efferocytosis, autophagy, inflammasome formation, and pyroptosis. These processes lead to lipid accumulation, proinflammatory activation, and foam cell formation in macrophages (Figure 1). Similarly, macrophage lncRNAs play a role in sepsis development by regulating inflammation, ROS generation, and M1 polarization (Figure 3). Pyroptosis and apoptotic cell death are crucial factors affecting sepsis progression [89,90], and macrophage lncRNAs that mediate these processes in sepsis are expected to be revealed soon. While many lncRNAs have been found to be transferred by exosomes in atherosclerosis, exosomal lncRNA has not been detected in conditions associated with sepsis. 

It is intriguing to observe that NEAT1 and MALAT1 serve as key regulators of macrophage functions in both atherosclerosis and sepsis. Despite their distinct target profiles, both MALAT1 and NEAT1 promote inflammation and facilitate M1 polarization or foam cell formation. When considering the roles of these two lncRNAs, it is important to acknowledge the presence of a series of tRNA-like transcripts generated from the evolutionarily conserved *NEAT1-MALAT1* gene cluster. Targeted disruption of these tRNA-like transcripts has revealed their critical involvement in the innate immune responses of monocytes/macrophages [91]. Exploring the interrelationship between these lncRNAs and tRNA-like transcripts in the pathogenesis of atherosclerosis and sepsis promises to be an intriguing avenue for future research.

In summary, manipulating the expression of numerous macrophage lncRNAs in experimental animal models has shown promising effects on the severity of atherosclerosis and sepsis. For instance, the introduction of MALAT1 siRNA effectively reduced macrophage-mediated inflammation and improved lung injury in a mouse model of LPS-induced sepsis [81,82]. Conversely, *MALAT1* overexpression was found to exacerbate disease severity in an atherosclerotic mouse model [43]. Furthermore, overexpression of *PCA3* or knockdown of KCNQ1OT1 in *apoE*^−/−^ mice demonstrated favorable outcomes by promoting reverse cholesterol transport and impeding atherosclerosis progression, whereas overexpression of *GAS5* exerted contrasting effects [36,38,40]. However, it is important to exercise caution when drawing conclusions solely based on lncRNA overexpression studies, as many lncRNAs may not be expressed at sufficiently high levels to function as effective miRNA sponges. Although clinical trial results are currently unavailable, the compelling findings from these animal studies underscore the potential of lncRNA-based therapy as a promising avenue for the treatment of atherosclerosis and sepsis. Further research and clinical investigations are warranted to explore the therapeutic implications and translate these findings into clinical practice.

## Data Availability

Not applicable.

## Abbreviations

ABCA1, ATP binding cassette subfamily a member 1; ADAM10, A disintegrin and metalloproteinase domain-containing protein 10; AI662270, Mus musculus expressed sequence AI662270; AKT, Protein kinase B; ANGPTL4, Angiopoietin-like 4; ANRIL, Antisense non-coding RNA in the INK4 locus; ATP, Adenosine triphosphate; BALF, Bronchoalveolar lavage fluid; BAZ1A, Bromodomain adjacent to Zinc finger domain 1A; BMDM, Bone marrow-derived macrophage; BTK, Bruton tyrosine kinase; C/EBPβ, CCAAT/enhancer binding protein beta; CAD, Coronary artery disease; cAMP, cyclic adenosine monophosphate; CD36, Cluster of differentiation 36; CD42, Cluster of differentiation 42; CD47, Cluster of differentiation 47; CDKN2B, Cyclin dependent kinase inhibitor 2B; CDKN2B-AS1, CDKN2B antisense RNA 1; COX-2, Cycloxygenase-2; CPT1, Camitine palmitoyltransferase I; CREB, cAMP response element-binding protein; CRNDE, Colorectal neoplasia differentially expressed; DDX17, DEAD-box helicase 17; Dnm3os, Dynamin 3 opposite strand; DNMT1, DNA(cytosine-5)-methyltransferase 1; ELAVL1, Embryonic lethal abnormal vision like RNA binding protein 1; EV, Extracellular vesicle; EZH2, Enhancer of zeste 2 polycomb repressive complex 2 subunit; FGF1, Fibroblast growth factor 1; FXR1, Fragile-X mental retardation autosomal homolog 1; GAS5, Growth arrest-specific 5; GPR, G-protein coupled receptor; GSEC, G-quadruplex-forming sequence containing lncRNA; H3K27, Trimethylation of lysine 27; HAND2, Heart and neural crest derivatives expressed 2; HAND2-AS1, HAND2 antisense RNA 1; HDAC3, Histone deacetylase 3; HMGB1, High mobility group box 1; HUVEC, Human umbilical vein endothelial cell; IFN-γ, Interferon gamma; IL-1, Interleukin 1; IL-1β, Interleukin 1 beta; IL-4, Interleukin 4; IL-6, Interleukin 6; IL-8, Interleukin 8; IL-10, Interleukin 10; IL-12, Interleukin 12; IL-13, Interleukin 13; INK4, Inhibitor of cyclin-dependent kinase 4; iNOS, inducible nitric oxide synthase; ITGB1, Integrin beta 1; KCNQ1, Potassium voltage-gated channel subfamily Q member 1; KCNQ1OT1, Kcnq1 overlapping transcript 1; KLF4, Kruppel-like factor 4; KLF5, Kruepeel-like factor 5; LAMP1, Lysosomal-associated membrane protein 1; LDL, Low-density lipoprotein; LncRNA H19, LncRNA encoded by the H19 gene; lncRNA, Long noncoding RNA; LncRNA-COX2, LncRNA located about 50kb upstream of the protein-coding gene COX-2; LPS, Lipopolysaccharide; LXR, Liver X receptor; m6A, N6-methyladenosine; MAARS, Macrophage-associated atherosclerotic lncRNA sequence; MALAT1, Metastasis-associated lung adenocarcinoma transcript 1; MAPK, Mitogen-activated protein kinase; MAS, Marker assisted selection or marker aided selection; MAT2A, Methionine adenosyltransferase 2A; Mdm2, Mouse double minute 2 homolog; MEG3, Maternally expressed 3; METTL16, Methyltransferase 16; MeXis, Macrophage-expressed LXR-induced sequence; MI, Myocardial infarction; MIAT, MI-associated transcript; miRNA, MicroRNA; MMP-9, Matrix metalloproteinase 9; MRGPRF, MAS related GPR family member F; MSC, mesenchymal stem cell; MyD88, Myeloid diffentiation primary response 88; ncRNA, Noncoding RNA; NEAT1, Shortened from either nuclear paraspeckle assembly transcript 1 or Nuclear enriched abundant transcript 1; NEXN, Nexilin F-actin binding protein; NEXN-AS1, NEXN antisense RNA 1; NFX1, Nuclear transcription factor X-box binding 1; NF-κB, Nuclear factor kappa-light-chain-enhancer of activated B cell; NLR, Nucleotide-binding domain leucine-rich repeat containing; NLRP3, NLR family pyrin domain containing 3; NONO, Non-POU domain containing octamer binding; oxLDL, Oxidized LDL; PBMC, Peripheral blood mononuclear cell; PCA3, Prostate cancer associated 3; PI3K, Phosphoinositide 3-kinase; Plin2, Perilipin 2; POU2F1, POU domain, class2, transcription factor 1; PPARα, Peroxisome proliferator activated receptor alpha PPARγ, Peroxisome proliferator activated receptor gamma PVT1, Plasmacytoma variant translocation 1; Rab, Ras-associated binding protein; RAB22A, Ras-related protein Rab-22A; RAPIA, LncRNA associated with the progression and intervention of atherosclerosis; RFX7, Regulatory factor X7; ROS, Reactive oxygen species; siRNA, Small interfering RNA; SIRT1, Sirtuin 1; SLAMF7, Signaling lymphocytic activation molecule 7; SMAD3, Small mothers against decapentaplegic homolog 3; SMILR, Smooth muscle-induced lncRNA; SNHG16, Small nucleolar RNA host gene 16; STAT3, Signal transducer and activator of transcription 3; TAK1, TGF-β-activated kinase 1; TGF-β, Transforming growth factor beta; TLR4, Toll-like receptor 4; TNF, Tumor necrosis factor; TNF-α, Tumor necrosis factor alpha; TRAF6, TNF receptor-associated factor 6; TUG1, Taurine-up-regulated gene 1; UCA1, Urothelial cancer associated 1; XIST, X-inactive specific transcript; ZFAS1, ZNFX1 antisense RNA 1; ZNFX1, Zinc finger NFX1-type containing.
